# 3D spheroid culture to examine adaptive therapy response in invading tumor cells

**DOI:** 10.1007/s44164-022-00040-x

**Published:** 2023-03-15

**Authors:** Felix Weiss, Nader Atlasy, Vince van Reijmersdal, Henk Stunnenberg, Cornelia Hulsbergen-Veelken, Peter Friedl

**Affiliations:** 1 Department of Cell Biology, Radboud University Medical Centre, P.O. Box 9101, 6525 GA Nijmegen, The Netherlands; 2 Department of Molecular Biology, Faculty of Science, Radboud University, 6525 AJ Nijmegen, The Netherlands; 3 David H. Koch Center for Applied Genitourinary Cancers, The University of Texas MD Anderson Cancer Center, Houston, TX 77030 USA; 4 Cancer Genomics Centre, 3584 CG Utrecht, The Netherlands

**Keywords:** 3D matrix culture, Spheroid, Cancer invasion, Resistance, Therapy response, Irradiation, Molecular profiling, RNAseq

## Abstract

3D in vitro culture models of cancer cells in extracellular matrix (ECM) have been developed to investigate drug targeting and resistance or, alternatively, mechanisms of invasion; however, models allowing analysis of shared pathways mediating invasion and therapy resistance are lacking. To evaluate therapy response associated with cancer cell invasion, we here used 3D invasion culture of tumor spheroids in 3D fibrillar collagen and applied Ethanol-Ethyl cinnamate (EtOH-ECi) based optical clearing to detect both spheroid core and invasion zone by subcellular-resolved 3D microscopy. When subjected to a single dose of irradiation (4 Gy), we detected significant cell survival in the invasion zone. By physical separation of the core and invasion zone, we identified differentially regulated genes preferentially engaged in invading cells controlling cell division, repair, and survival. This imaging-based 3D invasion culture may be useful for the analysis of complex therapy-response patterns in cancer cells in drug discovery and invasion-associated resistance development.

**Supplementary Information:**

The online version contains supplementary material available at 10.1007/s44164-022-00040-x.

## Introduction

The development and adaptation of 3D in vitro cell culture models as a platform for drug screening and to evaluate therapy response has advanced preclinical research by improving in-vivo relevance compared to classical 2D culture models [16]. Spheroid- or organoid-based cultures in 3D ECM, including reconstituted basement membrane and/or fibrillar collagen I, recapitulate aspects of the tumor microenvironmental ECM composition and stiffness as well as cell–cell and cell-ECM interactions [9]. 3D spheroid or tumoroid culture models have further been developed to reveal patterns and mechanisms of cancer cell invasion into ECM, a process relevant for initiating cancer metastasis [20]. Recent preclinical work suggested, that cell-ECM interactions involved in cancer cell invasion, including engagement of integrin adhesion receptors, also support DNA repair and cancer cell survival [5]. Invasion programs are further implicated in countering cell death, supporting metabolic adaptation, stemness, and survival mechanisms and, thus, contribute to therapy resistance in invading and metastatic cancer cell subsets [22].

We recently developed a 3D invasion culture model, which enables to study therapy response in invading cells [21]. Here, we improved the imaging capabilities by applying an optical clearing procedure, which allows quantification in deeper imaging planes and in the spheroid core. Additionally, we show that RNA sequencing techniques can be used for the identification of differentially regulated genes and pathways in the spheroid core and invasion zone.

## Methods

### Cell culture

Metastatic human melanoma cells (MV3) stably expressing H2B-GFP [21] or FUCCI sensor [15] were cultured in Dulbecco’s Modified Eagle Medium (DMEM; Gibco, 10,938) supplemented with 10% fetal bovine serum (FCS; Sigma, F7524), 100 U/ml penicillin and 0.1 mg/ml streptomycin (Pen-Strep; Sigma, P4333), 1 mM sodium pyruvate (Gibco, 11,360–039), and 2 mM L-Glutamine (Gibco, 25,030–024). The H2B-GFP signal was used for visualization of the nuclear chromatin, including nuclear integrity, and fragmentation [21].

### Spheroid formation

For the formation of spheroids, the hanging drop method was used. Cells at a high confluency (90%) were washed with PBS and detached with 2 mM EDTA (Invitrogen, 15,575,020) in PBS for 7 min at 37 °C. The lid of a 15 cm dish was filled with 100–120 droplets (30 µl per droplet including 5000 cells) of cell suspension containing culture medium, 4.8 mg/ml methyl cellulose (Sigma-Aldrich, M6385), and 10 µg/ml bovine dermis collagen I (PureCol, Advanced BioMatrix Inc., 5005). The dish was flipped and 5 ml of sterile PBS was added to the bottom dish to prevent dehydration. Subsequently, the dish was placed in the humidified incubator (37° C, 10% CO2) for 24 h. The concentric morphology of the spheroids, including circular shape, homogenous size distribution, compactness, and well-defined edges, was verified using bright-field microscopy, and spheroids were collected by gentle aspiration. Depending on the used cell type and experimental aim the concentration of methyl cellulose (2.4 or 4.8 mg/ml), spheroid size (500–5,000 cells/sph), droplet size, concentration of collagen, and duration of spheroid formation can be fine-tuned. Here, the higher concentration of methyl cellulose and the addition of collagen enhances MV3 melanoma cell aggregation. Compared to melanoma cells, epithelial cancer cells often require less methyl cellulose and collagen as aggregation enhancers because of their effective aggregation via adherens junctions.

### 3D invasion assay

The spheroids were washed three times with culture medium and stored in 0.5 – 1 ml of culture medium to be added to the collagen solution. Typically, 6 spheroids per experiment and condition were embedded 6 individual gels of non-pepsinized rat tail collagen I (Corning Inc., 354,249) solution (final concentration: 5 mg/ml). Collagen solution was prepared according to the manufacturer’s protocol (Alternate Gelation Procedure). Briefly, to obtain a total volume of 1.5 ml, 120 µl of 10 × PBS, 14.01 µl of 1 N NaOH (2% of collagen volume), and 283.86 µl of 80 mM HEPES buffer in MilliQ water were mixed and all steps were performed on ice. A volume of 782 µl of non-pepsinized rat-tail collagen type I stock solution (9.59 mg/ml) was added and mixed gently but thoroughly by pipette aspiration (15 s) to reach uniform distribution and pH within the solution, followed by the addition of 300 µl cell culture medium containing the spheroids. The collagen solution was carefully but thoroughly mixed by pipette aspiration and rolling the tube until a homogenous, interface-free aspect of the solution was reached. Per well (12-well plate), a single spheroid in the final collagen solution was identified by eye and picked up with a P200 pipette in a volume of 100 µl and added as a central drop. After filling of the complete plate with collagen drops, duplicate spheroids were removed with a P20 pipette. Next, the plate was incubated at 37 °C upside down for 90 s and then flipped every 60 s to avoid sedimentation of the spheroid until collagen polymerization was complete. Collagen polymerization was detected as gelification and milky appearance typically reached after 7 min. Afterincubation for further 30 min at 37 °C, cultures were overlaid with prewarmed medium (2 ml, 37 °C) and culture medium was replaced every 2–3 days upon consecutive long-term culture. The spheroids could be cultured for 7–10 days. Samples with 2D growth of cells between the well bottom and collagen gel were discarded.

### Irradiation of spheroid invasion cultures

After 2 days of culture, with invasion fully established, spheroid cultures were irradiated with a single dose of 4 Gray X-rays (X-RAD 320ix, at 3.08 Gy/min, 12.5 mA, 320 kV, 2 mm aluminum filter). Unirradiated samples were also carried to the radiation facility to account for the effect of temperature and gas fluctuations during transport.

### Sample staining and clearing

**Fig. 1:**
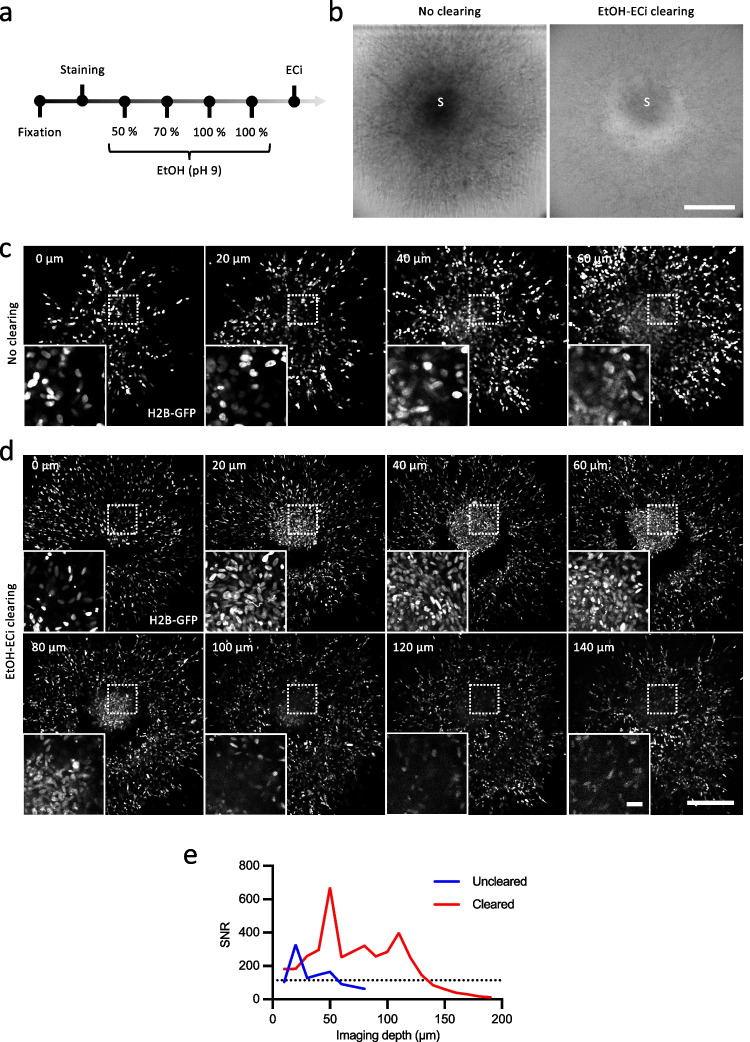
EtOH-ECI clearing of invading spheroids. **a** Steps of sample dehydration and refractive index matching by ECi. **b** MV3 spheroids (S) 2 days after embedding in collagen I without or with EtOH-ECi clearing imaged by brightfield microscopy. Subsequent (10 µm) z-slices from confocal microscopy without (**c**) and with clearing (**d**) showing H2B-GFP signal. Subcellular detail in insets. Scale bar, 200 µm. **e** Signal-to-noise ratio (SNR) as a function of imaging depth, from spheroids of panels (c) and (d). Dashed line indicates threshold for possible segmentation of nuclei

Spheroid samples in 3D collagen were fixed in 4% PBS-buffered paraformaldehyde (PFA) (30 min, 37 °C), washed twice with PBS-T (PBS supplemented with 0.1% Tween), and washed 3 further times with PBS (5 min, 20 °C). Samples were transferred to a 48-well plate using tweezers and stained with AlexaFluor568-phalloidin (Invitrogen, A12380; 330 nM) in 0.1% Triton X-100 (Sigma, T8787), 1% bovine serum albumin (BSA; Sigma) in PBS overnight (4 °C), washed 3 times with PBS (5 min, 37 °C) and twice with 0.05% NaN_3_ in PBS (5 min, 37 °C). For optical clearing of the samples, the Ethanol-ethyl cinnamate (EtOH-ECi) clearing protocol was adapted [7]. Samples were dehydrated with ethanol (800 µl) at increasing concentrations (50%, 70%, 100%; applied twice for 75 min at 4 °C) (Fig. 1A). EtOH solutions 50 and 70% were adjusted with sodium hydroxide (Sigma, 1310–73-2) to pH 9 at 4–8 °C. Samples were then transferred to 400 µl of ECi for refractive index matching (1.558) in polypropylene tubes (Micro Tube 1.5 ml, Sarstedt, 72.690.001) and incubated (75 min, 20 °C), after which ECi was refreshed (400 µl) for sample storage (20 °C; for up to a week). This procedure preserved the fluorescence of GFP and AlexaFluor568.

### Microscopic imaging

Samples were scanned in 3D by confocal microscopy (Zeiss LSM 880, Nikon Plan Apo 10 × /0.45 DICII objective, 2 × zoom). For quantification of invading cells in cleared samples, three z-slices at 10 µm steps were acquired from the spheroid center, defined as the z-plane with a maximum diameter of the rim of the core. For representative quantification, at least one quadrant of the spheroid perimeter and invasion zone were imaged as a 3 × 3 tilescan. For quantification of the core and rim, the entire spheroid was imaged with z-slices at 6 µm steps and a total depth of 360 µm.

### Image analysis

Images were processed using Fiji (2.3.0/1.53q) [17]. The boundary between the spheroid rim and invasion zone was defined manually, based on the transition from compact cell organization towards a phase of individualizedcell bodies and polarization of cells towards the invasion front, using the polygon selection tool and Fit Ellipse command to straighten the selection outline. The boundary between the core and rim was defined by the transition from compact ring to the cell-rich mass inside the hollow core. The region of automated single-cell segmentation in the invasion zone was defined as one random quadrant originating from the centroid of the spheroid core. For analysis of the core the inside region of the rim was used and for the rim the inside and outside boundaries of the rim. Segmentation of cell nuclei (H2B-GFP channel) was performed for the core and the invasion zone separately. The images were pre-processed by applying a Gaussian blur filter (σ = 1.5 scaled). To account for the heterogeneity of signal intensity and contrast caused by variable z-position of nuclei, local adaptive thresholding (Auto Local Threshold, Phansalkar method [13]) was performed, followed by Median filtering (radius = 3) and separation of adjacent nuclei (Watershed function). Nuclei were segmented using the Particle Analyzer (size 45–700 µm). The absolute number of nuclei in each slice in the core or invasion zone and their average per slice were quantified. The distance from each segmented nucleus to the centroid of the spheroid was calculated to estimate the distance migrated by each cell. Fragmented nuclei were detected as small fragments (3–25 µm^2^) of the segmented H2B-GFP event within 20 µm radial distance using the Automated Cellular Analysis System (ACAS, MetaViLabs, Austin TX, USA) [21]. For MV3 cells after irradiation, fragmentation of the nuclei after irradiation is associated with the induction of activated caspase-3 and, hence, considered apoptotic [21]. Mitotic events were counted manually, based on condensed chromosomes of variable positioning with high fluorescent intensity in the H2B-GFP channel combined with the lack of a nuclear contour. Signal-to-Noise ratio (SNR) as function of imaging depth was calculated for each slice from the mean intensity of the upper 5% of the histogram (*I*_*mean*_). In a dark region mean intensity (*B*_*mean*_) and standard deviation (*SD*_*mean*_) of the background were measured. The SNR was calculated as (*I*_*mean*_*-B*_*mean*_)/*SD*_*mean*_. 3D reconstructions and lateral views were generated with Imaris (9.9.1).

### Image-guided dissection of 3D spheroids

MV3 H2B-GFP spheroids with a diameter of 1 mm (50,000 cells) were cultured for 2 days. For RNA isolation and RNA sequencing, approximately 10fold higher numbers of cells were required, compared to samples used for sample clearing and 3D microscopy. After replacing the medium by PBS, samples were transferred to a sterile fluorescence stereomicroscope for image-guided dissection (Leica MZFLII, 0.125 NA., EL6000external light source, GFP Plus filter sets). PBS was removed and the core was punched out using a 1 mm biopsy puncher (Miltex, Sterile Biopsy punch with plunger, #3331AAP/25) by pressing the punch down around the spheroid core and sucking it out using the punch plunger. The core sample was released into a sterile 1.5 ml Eppendorf tube and immediately placed on dry ice for snap-freezing. The corresponding invasion zone was controlled for any left-over core region, recognizable by much brighter fluorescence, and removed from the collagen gel with a 3 mm biopsy puncher (Miltex, Sterile Biopsy punch with plunger, #3332P/25). Exclusion criteria were ellipsoid-shaped spheroids, unsuccessfully punched spheroids, and ruptured collagen gels. Samples were stored at − 80 °C until RNA extraction.

### RNA sequencing

RNA was isolated using an RNA isolation kit (Qiagen, RNeasy Micro Kit) according to the manufacturer’s protocol. RNA concentration was measured using a NanoDrop 2000 (ThermoFisher) and stored at − 80 °C. The integrity and quality of RNA were evaluated using the Experion RNA HighSens AnalysisKit (Biorad, 7,007,105) on a 2100 Bioanalyzer platform (Agilent). For CDNA synthesis starting from 1 to 10 ng total RNA, SMARTer Ultra low Input RNA kit (Clontech, 634,936) was used and processed according to the manufacturer’s protocol. The transcribed DNA was sheared into fragments of 200–600 bp using Bioruptor Pico sonication device (Diagenode). Subsequently, the library preparation for Next Generation Sequencing has been carried out according to the Illumina standard protocol using the KAPA Hyper prep kit (KAPA Biosystems). Raw FASTQ data was cleaned from ribosomal RNA using Bowtie [10] and the sequencing reads with quality score higher than 15 were chosen for subsequent analysis. Processed reads were aligned to the human genome (hg38) using STAR aligner with default parameters [2]. Human uniquely mapped reads were annotated and counted against the human genome using HTSeq-count [1]. The data table was constructed based on the empirical counted reads. Subsequently, to obtain the differentially expressed genes between conditions, size factor normalization and pair-wise negative binomial test were used within the DESeq2 package [11]. Genes with expression difference of twofold change and *p*-value < 0.05 were considered to be differentially expressed. Heatmaps of variable genes were created with ComplexHeatmap package [4]. The enrichR package for R was used for the database query and visualization of the enrichment results [8].

### Statistics

Statistical analysis was performed using GraphPad Prism version 9.4.1. *P*-values smaller than 0.05 were considered significant.

## Results

### Monitoring invasion and therapy response in 3D spheroid culture

To quantify invasion and therapy response in 3D spheroid culture in all compartments, including (i) the core, (ii) the margin to the collagen matrix (rim), and (iii) the invasion zone, microscopic imaging with single-cell resolution reaching the depth of the spheroid core was performed. Cell detection inside the core is hampered by light scattering [21]. In native samples scanned by confocal microscopy, without optical clearing, tumor cell nuclei were reliably detected within 20–40 µm of imaging depth from the upper spheroid border (Fig. 1c). To overcome light scattering and absorption by the 3D sample, refractive index matching by the EtOH-ECi protocol was applied (Fig. 1a), which resulted in efficient optical clearing (Fig. 1b) and reduced signal scattering at higher imaging depth (Fig. 1d). Clearing improved the SNR and allows more than two-fold imaging depth compared to uncleared samples (Fig. 1e).

The ECi procedure results in notably decreased fluorescence intensity of fluorophores and fluorescent proteins due to the sample preparation steps [12]. Fluorescence can be preserved by shortening the duration and decreasing the temperature of dehydration (4 °C) as well adjustment of EtOH-water solutions to pH 9 to decrease the destabilization of fluorescent probes/proteins [14]. Optical clearing protocols are still being optimized and should be adapted to the experimental requirements. For example, alternative dehydration agents can be considered, e.g., using tert-butanol or 1-propanol [12, 19].

### Analysis of therapy response

**Fig. 2:**
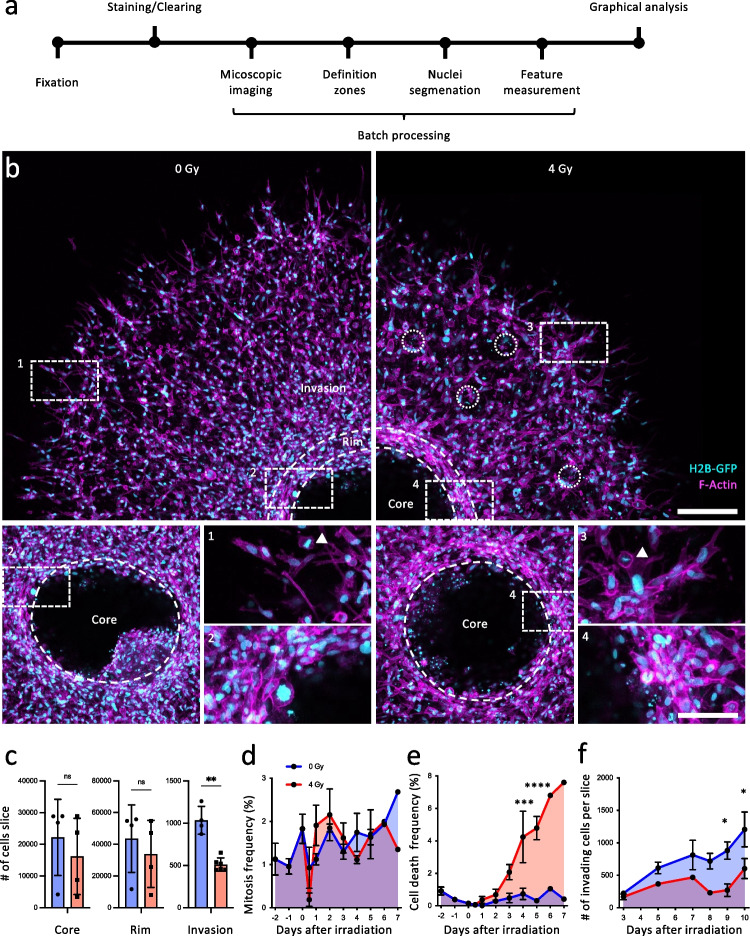
3D microscopy and analysis of invading cancer cells. **a** Timeline of image acquisition and analysis. **b** MV3 spheroids at the endpoint on day 7 of rat-tail collagen I culture after irradiation with 0 or 4 Gy irradiation on day 2. Z-projection of three slices with interslice distance of 10 µm. Circles indicate apoptotic events. Arrowheads indicate mitosis. Scale bar, 200 µm (overview), 65 µm (inset). **c** Absolute number of cells in the complete core and rim, and in three central slices of the invasion zone 7 days after embedding with 0/4 Gy irradiation on day 2. *n* = 4–6 spheroids. **d** Mitosis frequency and **e** cell death frequency over time. *n* = 15 spheroids. **f** Absolute number of invading cells in the invasion zone over time with 0/4 Gy irradiation. *n* = 2–4 spheroids. *p < 0.05, **p < 0.01, ***p < 0.001, *p < 0.0001; where significance is not indicated *p* > 0.05. Mann–Whitney test was used for panel c and two-way ANOVA for d, e, and f

Confocal imaging of cleared samples allowed to detect the effect of irradiation (single dose, 4 Gy) on cell invasion and survival at single cell level (Fig. 2a, b). Within 7 days of 3D spheroid culture, cells disseminated up to 850 µm into the collagen matrix (Fig. 2b). Concurrently, the initial volume of spheroid was near-completely depleted of cells, with a small cell-dense residual tumor mass (core) connected to the concentric rim along the previous spheroid-collagen interphase **(**Fig. 2b; Suppl. Figure 1). The invading cells were polarized with long actin-rich protrusions in radially orientation from the spheroid center and gradually decreasing cell density towards the invasion front (Fig. 2b).

After irradiation, the number of cells in the invasion zone was reduced by 50% and the invasion distance was reduced by approximately 100 µm, compared to sham-treated cultures (Fig. 2b, c). The frequency of mitosis by day 7 (5 days post-irradiation) was comparable between both conditions (Fig. 2b, d). However, in the invasion zone the frequency of cells in S/G2/M-phase compared to the core was increased (Suppl. Figure 2). The frequency of cell death, detected as fragmentation of nuclei, which ranges below 1% in the invasion zone of control cultures at early time points, was increased to 8%, when monitored 7 days after irradiation (Fig. 2e). Irradiation neither affected the residual cells located in the previous spheroid core nor the cell-dense rim, whereas the cell number in the invasion zone was decreased by 50% (Fig. 2b, c). However, a major subset of invading cells survived and even regained the ability to proliferate 10 days after irradiation (Fig. 2f). Whereas the effect of single-dose irradiation in the invasion zone was reliably detected, the effects on the spheroid core were non-conclusive, due to cell depletion under control conditions after cumulative 7 days of culture, which is a physiologically irrelevant phenomenon compared to intact tumors which lack regions of cell depletion.

### Differential transcriptional regulation in core and invasion zone

**Fig. 3:**
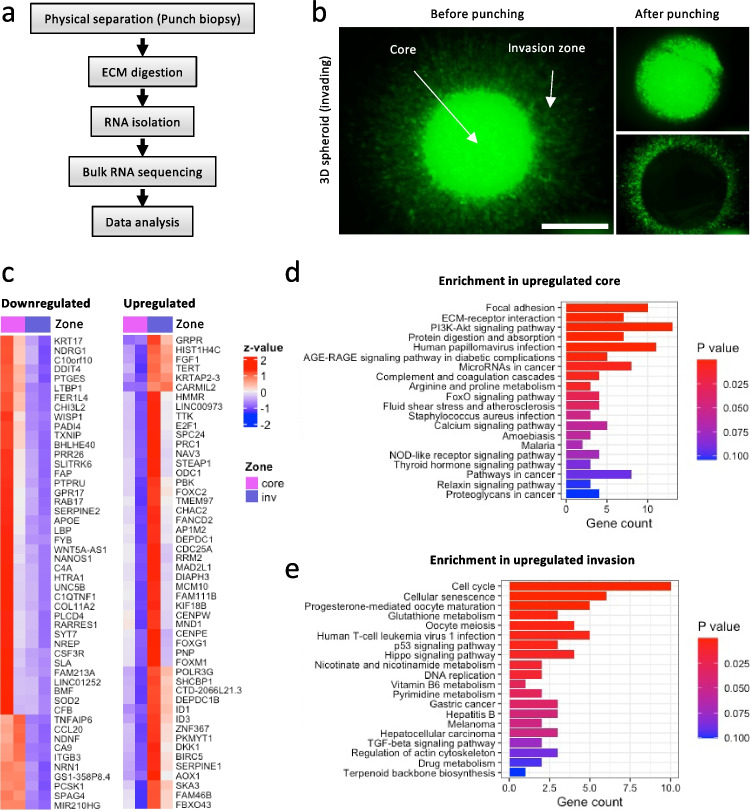
Differential gene regulation in invading cancer cells. **a** Flow chart of sample processing for cell isolation and RNA sequencing. **b** MV3 spheroids 2 days after embedding in collagen I before and after separation with 1 mm biopsy punch imaged by binocular microscopy. Scale bar, 700 um (**c**) Down- and upregulated genes in invading cells compared to the non-migratory core 2 days after collagen I embedding, *n* = 2. Enriched KEGG pathways for downregulated (**d**) and upregulated genes (**e**) in invading cells

To explore the respective differential signaling programs present in the spheroid core and invasion zone, we performed image-guided microdissection of both zones 2 days after spheroid embedding and performed bulk RNAseq (Fig. 3a). The core, which showed no signs of cell depletion at this time point, was physically isolated by punch biopsy, with the intact invasion zone remaining in the collagen environment (Fig. 3b). After cell harvesting and differential RNA expression analysis, 96 upregulated and 174 downregulated genes between differentially expressed core and invasion zone (Fig. 3c). The downregulated genes were found to be enriched in the *focal adhesion* (PDGFRB, COL1A1, MAPK10, LAMA5, ITGA10, ITGB3, COL6A2, COL6A1, FN1, VEGFA), *ECM-receptor interaction* (COL1A1, LAMA5, ITGB3, COL6A2, ITGA10, COL6A1, FN1), PI3K-Akt (PDGFRB, LAMA5, CSF3R, ITGB3, FN1, VEGFA, COL1A1, EFNA1, BCL2L11, ITGA10, COL6A2, DDIT4, COL6A1), and *FoxO signaling* (MAPK10, BCL2L11, CCNG2, SOD2) pathways. Among the top pathways enriched in the upregulated genes, *cell cycle* (CCNA2, ORC6, CDC45, E2F1, BUB1B, MCM5, TTK, PKMYT1, CDC25A, MAD2L1), *cellular senescence* (CCNA2, SERPINE1, E2F1, MYBL2, FOXM1, CDC25A), *p53 signaling* (RRM2, SERPINE1, GTSE1), and *Hippo signaling* (SERPINE1, ID1, BIRC5, FGF1) were found (Fig. 3d). The upregulation of cell cycle-related genes correlated with increased frequency of invading cells in S/G2/M-phase compared to non-invading cells (Suppl. Figure 2). Thus, significant gene expression regulation of cell-cycle- and survival-associated pathways was detected in the invasion zone of collagen-based 3D spheroid culture.

## Discussion

Optical sectioning of focal planes within light-scattering 3D specimens is enabled by EtOH-ECi-based optical clearing. This protocol retains most of the fluorescence of many fluorophores and fluorescent proteins and further does not involve harmful or corrosive chemicals [7]. The ECi-clearing procedure takes only hours and can therefore easily be implemented in workflows with broad applicability in routine laboratory environments and microscopy facilities. In the future, optimization of dehydration solvents (e.g., isopropanol) [12] will increase fluorescence retention. ECi-resistant 48 well plates are currently not commercially available and storage in those instead of Eppendorf tubes is desired for faster sample handling. Here, cancer cells stably expressed the reporter gene H2B-GFP for the visualization of nuclear integrity or fragmentation without further need of nuclear labeling, as described [5]. In addition, EtOH-ECi clearing can be combined with phalloidin and antibody labeling, which ensures broad applicability towards investigating proteins and structures of interest [7]. The refractive index matching of ECi precludes the detection of collagen fibers by reflection imaging, however fluorescently pre-labeled collagen or collagen-specific antibody staining for ECM visualization might be used [3]. Mitosis, cell death, cell number, and morphology can be studied and combined with specific labeling strategies to further study proteins of interest.

MV3 melanoma cells are highly migratory and emigrated effectively from the initial spheroid, resulting in near-complete cell depletion of the spheroid core. With the emergence of hollow zones in the spheroid core, which can be considered as non-physiological consequence of cell exhaustion due to excessive invasiveness in the in vitro model, the physiological relevance of therapy effects on the tumor core may be limited.

Similar to in vivo outcomes, where invading cancer cells gain integrin αV/β1 dependent resistance to radiotherapy [5], we aimed to recapitulate invasion-associated acquired radiation resistance in long-term 3D culture model and detected tumor cell survival 5 days post-radiotherapy. Collagen may represent a minimal tissue equivalent to model integrin-dependent and other pathways of invasion associated cell survival and therapy resistance [22].

Enrichment of non-migratory and invading cancer cells allows to investigate differential regulation by next-generation sequencing techniques (e.g., RNAseq and single-cell RNAseq). Separation of the core requires larger spheroids in culture (10 × higher cell number compared to spheroids in invasion culture). This size difference may alter the signaling states in invading cells or the core, and therefore potentially affect the match between transcriptomic analysis and functional readout in invasion culture. Furthermore, the zonal separation was performed by vertical dissection, thus contamination of invading cell subsets at apical and basal poles of the culture may be retained in the core biopsy. In addition to bulk sequencing, performed here, invading spheroids could be molecularly profiled by single-cell RNA sequencing. The zones or subpopulations could then be separated virtually by graph-based clustering approaches [23]. Invasion-associated regulation of cell cycle, cellular senescence, and hippo signaling may be associated with cell adhesion signaling and altered mechanotransduction in invading cells, including signaling through integrins, FAK, ILK, YAP, and other pathways [6, 18, 22].

As a minimal tissue-equivalent 3D culture model, this assay can be used to investigate the invasion-specific response to treatment and is suitable for functional drug screening. Enriched pathways indicate that invading cells alter their molecular programs which not only may affect cell motility but also cell division, repair, stemness, and survival signaling. This is in line with altered therapy response in invading tumor subsets and underlines the importance to study shared mechanisms of invasion and resistance signaling [22]. The 3D spheroid-based molecular characterization by bulk or single-cell RNA sequencing offers insights in differential regulation in invading cancer cells upon treatment and can therefore be used as discovery tool.

## Supplementary Information

Below is the link to the electronic supplementary material.

Supplementary file1 (PDF 0.98 MB)

## Data Availability

The RNAseq datasets generated during the current study are available in the GEO repository (GSE215750). Image analysis macros are available in the GitHub repository (https://github.com/ftoenisen/3D_invasion_therapy_response).
