# Supplementary material for: 3D spheroid culture to examine adaptive therapy response in invading tumor cells
Source: In Vitro Model. 2023 Mar 15;1(6):463–71. doi: 10.1007/s44164-022-00040-x (PMC10119213; doi:10.1007/s44164-022-00040-x)
Supplement: Supplementary file 1 — Supplementary file1 (PDF 0.98 MB) [file 44164_2022_40_MOESM1_ESM.pdf]

Supplementary Figure 1

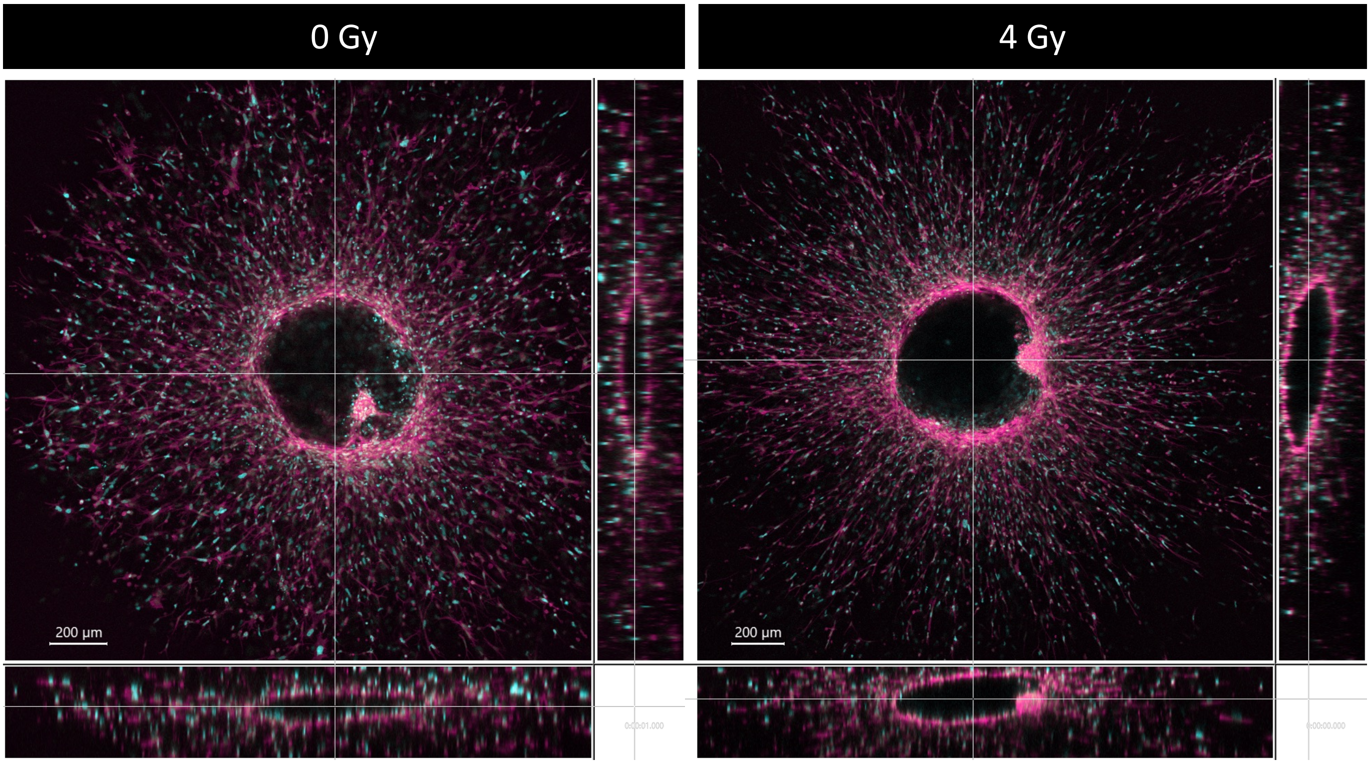

Supplementary Fig. 1 3D reconstruction of invading spheroids
3D reconstruction and lateral views of MV3 spheroids at the endpoint on day 7 of rat-tail collagen I culture after irradiation with 0 or 4 Gy irradiation on day 2. cyan = H2B-GFP, magenta = F-actin.

Supplementary Figure 2

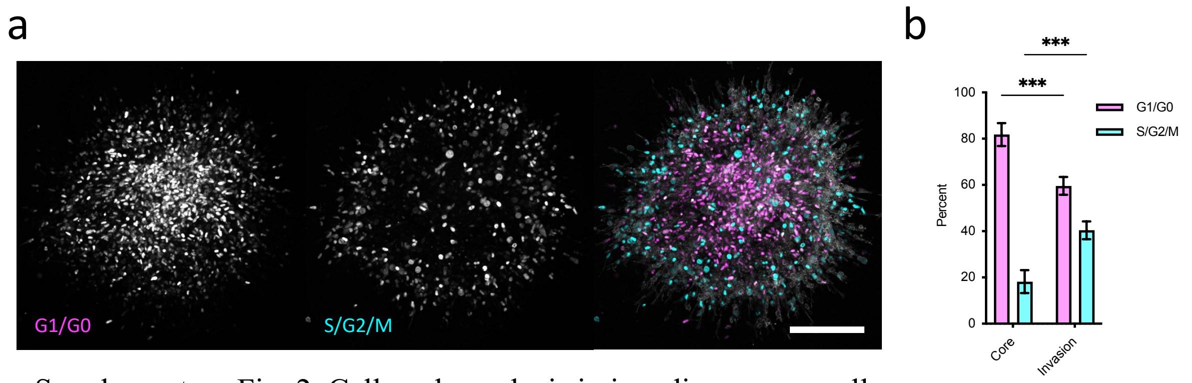

Supplementary Fig. 2. Cell cycle analysis in invading cancer cells
a) MV3 spheroids expressing FUCCI sensor 2 days after embedding in collagen I. b) Frequency of cells in G1/G0 and S/G2/M phase per zone. n = 15 spheroids. Scale bar, 200 µm. Two-way ANOVA was used for the statistical analysis. ***$p<0.001$.
